# The Medical Management of Consumptives

**Published:** 1912-10-19

**Authors:** 


### SANATORIA: THEIR USE AND ABUSE.

(Contributions, experiences and questions relating to Sanatoria, their Administration and Work, are invited.)

### The Medical Management of Consumptives.

BY A SANATORIUM SUPERINTENDENT.

The well-known facial aspect of "the lords of human kind" was ever displeasing to revolutionary youth, by which last two words the medical student used to be fairly well described. I say "used" because in the last fifteen years a change has come over this individual. Formerly he was one of those who read George Gissing with a sympathetic thrill and listened eagerly to elders when they talked about Bradlaugh. But now, just as that fine observer Mr. Shaw points out in the case of the private soldier, who no longer resembles Mr. Kipling's three heroes, but is rather "a depressed and respectable young man," so our budding physician is becoming a conventional member of the upper middle-class, who is careful to put on dark clothes when he goes calling on Sundays and likes testimonials from bishops. Which of the two types, the old or the new, is the better is a question upon which it would never do to enter; but as to which of them will adapt himself more easily to the ways of the world there can be no dispute at all. At first, at any rate, the authoritarian-trained product will get the halfpence and his anti-type the kicks. Yet still, in spite of the change mentioned, some young medical men begin their professional careers with naïveté enough to think that patients really desire reasoned explanations and not orders—moderate, temperate, kindly expressed, well-thought-out orders, but still orders—the expression of will which makes a man succeed with women and horses. And even one with plenty of firmness, if it is required, may dislike (and rightly) all the prudent shows of authority—the pursed lip, the rather pompous pose, the attention to appearance, the slight chilliness and reticence, all the manifold little insincerities which everywhere characterise the one in authority, unless, being a remarkable man, he has prestige enough not to need them. Idealists must pick up by experience the regulation equipment of behaviour necessary to secure obedience. Those who say that public-school training is a prerequisite cannot have noticed very much. Any good foreman or non-commissioned officer knows all there is to learn about it.

In matters medical obedience from the patient is most easily secured when it concerns something highly technical. Any reasonable adult will obey one over taking medicine, for instance, although to swallow down a draught on the instant is a strong expression of confidence. About the medicine he knows that he knows nothing, and recognises that he has therefore no grounds for argument against it. It is a matter for the technical expert alone, who must be trusted altogether or not at all. This is one reason why, although our great hospitals receive some pretty rough customers, obedience is always secured there. There are other reasons, too. One is that the patients are mostly acutely ill, and therefore in low physical condition and in some fear of pain, disablement, or death, to lessen which risks they will submit to much. Yet even so, mark how our nursing system provides aids to securing obedience. In every hospital there is attention to appearance—the meticulous spick and spanness. There are the religious services bringing solace to true believers, and thus fulfilling, like the last, other purposes as well, but still just as actually upholding authority as the Church did in Voltaire's day and has done since.

Now mark again how all these conditions favouring obedience are wanting in the case of the sanatorium. The inmates are seldom acutely ill; very soon they get stout and jolly. The breezes of Heaven, the open-air life, and tramping in and out of muddy boots are impossible to combine with polished floors, shining brass, and a palm covered ward table. Besides, the upkeep of appearance costs money, and sanatoriums, up to now the Cinderellas of the charitable world, are much poorer than hospitals, while to provide all the additional beds required will absorb all the available funds in bare necessities without much doubt. As to services, singing to a harmonium two or three times a day would make the patient cough too much, and, anyhow, is not to the taste of the ordinary Anglo-Saxon so soon as he ceases to be bedfast. "When the devil was sick . . ."—that is the whole point, that the "lunger" so soon forgets that he *is* sick.

In addition it has to be remembered that there is talk of a special psychology of the consumptive—whether congenital or tuberculo-toxic does not affect our present purpose—characterised by traits not conducing at all to manageableness. All that is *the first requisite of the sanatorium medical superintendent is not high degrees, not kindness and sympathy at all, but the power to secure obedience;* if by the prestige arising from intellectual endowment so much the better. Only one qualification need be made to the above, which is that it is assumed he knows what the hygienic-dietetic treatment of consumption is in actual practice, from residence in, not merely a visit to, a good sanatorium. There is no doubt that in the past such a sanatorium would be of the Nordrach type. It is surprising how little, really, subsequent experience has added to what Walther taught. We know now that for walking exercise manual labour can be partly substituted without great therapeutic loss and with great economic gain. In two or three years we shall know as certainly, probably, that tuberculin on the intensive system is a help. But the groundwork—of reliance on indications furnished by the thermometer—remains essential and can only be learnt from resident experience. There are highly placed physicians who, from lack of this resident experience, can diagnose the disease, but hardly treat it. And just as, half unconsciously, our hospital matrons insist on hymn-books and brass-rags (*laborare est orare*—it is an old collocation after all), just so, half subconsciously too, did Walther magnify most effectively the office of the resident medical man. He has not been quite forgiven for this yet, although Dr. Paterson has since arisen to show how right he was. To the above arguments the only cogent objection the writer can perceive is the one that, like the French jeweller, he is an interested party. Well, let the following plain tale put this objection down.

A certain young *locum tenens* appeared on the scene at a sanatorium. He was highly conscientious, and kept the charge nurse afoot to a late hour on his evening rounds. But his manner was altogether too conciliatory. It may have pleased certain of the married women with its sympathy, but in a week he was known privately among the patients as "Gentle Annie." In a fortnight those who fancied they could walk farther than he sent them, or found their rest hour tedious, began to argue these points with him, often successfully. In fact, as they say at sea, the ship began to take charge, and the medical situation would soon have come to resemble that prevalent at certain hotels, where a visiting doctor is provided as a diversion by the Thelemite management, to be heeded or disregarded by the guests at their own sweet wills. It is one of the advantages of the central administration of medical benefit now impending that patients will be protected against themselves. In the management of the consumptive, where so much is at stake and where therapeutic resources are so slender, there is no room for indulgence to arbitrary individual predilections on the part of the patient. It is a point which the ordinary layman, with mistaken kindness, does not perceive, and which, therefore, at the present juncture needs plainly pointing out.

